# Long-Term Corticosteroid-Induced Ocular Hypertension Model Produced by Topical Rinderon-A Ointment Application

**DOI:** 10.3390/biology15151289

**Published:** 2026-08-04

**Authors:** Hsin-Lin Cheng, Yung-Chang Yen, Jiann-Jou Yang

**Affiliations:** 1 Department of Biomedical Sciences, Chung Shan Medical University, Taichung 40201, Taiwan; 0003018zoommuzik@gmail.com; 2 Department of Ophthalmology, Chi-Mei Hospital Liou-Ying, Tainan 73657, Taiwan; 3 Department of Nursing, Min-Hwei Junior College of Health Care Management, Tainan 73658, Taiwan; 4 Department of Medical Research, Chung Shan Medical University Hospital, Taichung 40201, Taiwan

**Keywords:** ocular hypertension, corticosteroid, Rinderon-A ointment, rat model, intra-ocular pressure

## Abstract

Adverse effects associated with prolonged corticosteroid therapy are strongly associated with increased intraocular pressure (IOP), which may progress to iatrogenic glaucoma and irreversible blindness if left untreated. Developing reliable animal models is essential for investigating corticosteroid-induced ocular hypertension and evaluating potential therapeutic strategies. In this study, we established a simple and non-invasive rat model of corticosteroid-induced ocular hypertension using topical application of Rinderon-A ointment. Longitudinal IOP measurements were performed weekly using a Tono-Pen in conscious rats throughout the 11-week experimental period. Rinderon-A treatment resulted in sustained bilateral IOP elevation, with an initial increase observed at week 1 (28.4 ± 1.5 mmHg) and a maximal mean IOP of 31.2 ± 0.7 mmHg at week 10. Rinderon-A-treated eyes exhibited a sustained ocular hypertensive response throughout the experimental period. Collectively, this model provides a reproducible platform for studying corticosteroid-associated ocular hypertension and may facilitate future investigations of therapeutic interventions.

## Simple Summary

Prolonged corticosteroid therapy is strongly correlated with intraocular pressure (IOP) elevation and irreversible blindness. To evaluate potential therapeutic interventions, developing a reliable preclinical animal model to investigate the pathogenesis of ocular hypertension is crucial. This study established a non-invasive, highly reproducible rat model using topical Rinderon-A ointment. Over a 10-week experimental period, the subjects exhibited significant and sustained IOP elevation, demonstrating a 100% disease occurrence rate without requiring invasive surgical procedures. This stable and standardized animal model provides an excellent preclinical screening platform for assessing the efficacy of novel anti-glaucoma therapies by effectively mimicking chronic corticosteroid-induced ocular responses in humans.

## 1. Introduction

Glaucoma is generally accepted as the major cause of irreversible life-altering vision loss and degenerative nerve disease, which was characterized by loss of retinal ganglion cells (RGCs) [1]. Although the disease mechanism of glaucoma remains unclear, it is recognized that elevated IOP is the critical factor with the disease risk [2,3]. In addition, other pathological factors such as dysfunctional mitochondria, excessive oxidative stress, and blood-flow abnormalities were involved in the development of glaucomatous optic neuropathy [4,5]. The current efforts mainly focus on exploring the glaucoma disease progression and developed novel therapeutic options, while much remains to be elucidated, especially prolonged eye pressure elevation. Among secondary glaucoma, corticosteroid-induced ocular hypertension represents an important clinical complication associated with prolonged glucocorticoid therapy and may subsequently progress to steroid-induced glaucoma in susceptible individuals. Approximately 40% of individuals receiving corticosteroid treatment may develop corticosteroid-induced ocular hypertension, indicating substantial interindividual variability in corticosteroid responsiveness [6,7]. However, the mechanisms underlying corticosteroid susceptibility and ocular hypertension development remain incompletely understood.

New strategies involving tremendous studies and treatment information of glaucoma required a reliable, inducible, and easy-to-use platform for laboratory investigation of pre-clinical evidence that mimics possible pathological conditions of human glaucoma. Recently, animal models of artificially induced elevated IOP were classified into an inherited spontaneous model and venous outflow dependent model, which was shown to exhibit significant damage in the RGC and optic nerve [8,9]. A variety of animal models that had been built on mammal species including monkeys, rabbits, dogs, and mice were described in earlier studies [10,11,12]. In general, rodents served as the most popular models of ocular hypertension on the basis of functional similarity in anatomical and developmental abnormalities with humans, particularly in IOP elevation, which caused aqueous humor dynamics and optic nerve damage [13,14,15]. Thus, rodent species provided appropriate systems for non-primate experimental models with the advantages of low cost and being easy to utilize, which enabled us to perform large-scale in vivo studies. Several methods have developed to induce IOP elevation such as hypertonic saline injection, venous obstruction, and laser photocoagulation [16,17,18]. Each approach included an untreated control eye and an eye that had been treated by ablating the trabecular meshwork and injecting viscous material and microbeads [19]. In addition to surgically induced ocular hypertension models, steroid-induced ocular hypertension models have been widely used to investigate corticosteroid-associated IOP elevation and to evaluate potential therapeutic interventions. Previous studies have established steroid-induced ocular hypertension models using different corticosteroid formulations and administration approaches in mice, rats, and rabbits [20,21,22,23].

Despite the availability of several experimental models of ocular hypertension, many existing approaches involve invasive procedures or technically demanding methodologies, which may limit reproducibility and broader applications. Although steroid-induced ocular hypertension models have improved the investigation of corticosteroid-associated ocular hypertension, variations in induction protocols and IOP response profiles highlight the need for further model optimization and characterization. To address this unmet need, the present study aimed to establish a rat model of steroid-induced ocular hypertension through topical administration of Rinderon-A ointment. This model provides a simple and reproducible approach for longitudinal IOP monitoring without surgical intervention and may serve as a useful tool for preclinical studies of steroid-induced ocular hypertension.

## 2. Materials and Methods

### 2.1. The Establishment of an Animal Model

Ten male Wistar rats (200–300 g) aged six weeks old were purchased from BioLASCO Taiwan Co., Ltd. (Taipei, Taiwan) and maintained in the animal facility of Chi Mei Medical Center, under a controlled light condition (12–h light/dark cycle) in a 23 ± 2 °C (60% relative humidity) constant environment with free access to chow and water. All the experimental animal procedures involved in the study were reviewed and approved by the Institutional Animal Care and Care Committee of Chi Mei Medical Center (protocol code: 98010511. Date: 5 January 2009). Wistar rats were adaptively fed a laboratory control diet for 1 week and then assigned to three groups using a computer-generated random sequence (Microsoft Excel) to minimize selection bias: (i) both eyes of rats received topical application of Rinderon-A ointment (*n* = 5), (ii) the right eye received topical application of Rinderon-A ointment and the left eye received normal saline (*n* = 3), and (iii) both eyes of rats received topical application of normal saline (*n* = 2). During the experimental period, normal saline (internal control) or Rinderon-A ointment (betamethasone disodium phosphate, 1 g per tube) was topically applied to the eyes once daily for 11 weeks to induce ocular hypertension. At each administration, a thin layer of ointment was gently applied to completely cover the corneal surface. Throughout the experimental period, animals were monitored daily for general health status and signs of ocular irritation or distress. To prevent measurement bias, all IOP measurements and data analyses were conducted by an independent investigator who was strictly blinded to the group assignments.

### 2.2. Measurement of Intra-Ocular Pressure

In rats, IOP of each eye was measured 2 weeks before and after induction by using Tono-Pen XL (Mentor, Norwell, MA, USA), as previously described [24]. To reduce agitation, the rats were anesthetized with intramuscular injection of ketamine (45 mg/kg)/xylazine (9 mg/kg), and IOP measurement was started 5 min after anesthesia. Baseline IOP values were determined two weeks before Rinderon-A ointment induction and then were recorded once per week for eleven consecutive weeks post induction. IOP for each eye was measured at the same time of day (morning, 9–12 AM) and under a bright operating environment. For each procedure, five measurements were repeated to obtain individual reads and the average IOP readings of each eye were used for the final analysis.

### 2.3. Statistical Analysis

Data are presented as mean ± SD. Longitudinal IOP changes over the 11-week treatment period were evaluated using a two-way mixed-effects model (REML) with Geisser–Greenhouse correction to account for repeated measurements and unbalanced cohort sizes, followed by Bonferroni’s post hoc multiple comparisons test. A post hoc power calculation was conducted using G*Power software (version 3.1.9.7) based on the primary longitudinal outcome (IOP elevation). Given the large effect size (Cohen’s f > 0.80) observed between the treatment and control groups, the achieved statistical power (1-β) exceeded 80% (α = 0.05) despite the small cohort size (N = 10). All statistical tests were two-tailed, with statistical significance defined as *p* < 0.05. All analyses and plotting were conducted using GraphPad Prism (version 8.4.2, GraphPad Software, San Diego, CA, USA).

## 3. Results

### Induction of IOP Elevation in Rinderon-A Ointment-Treated Rat

**Figure 1:**
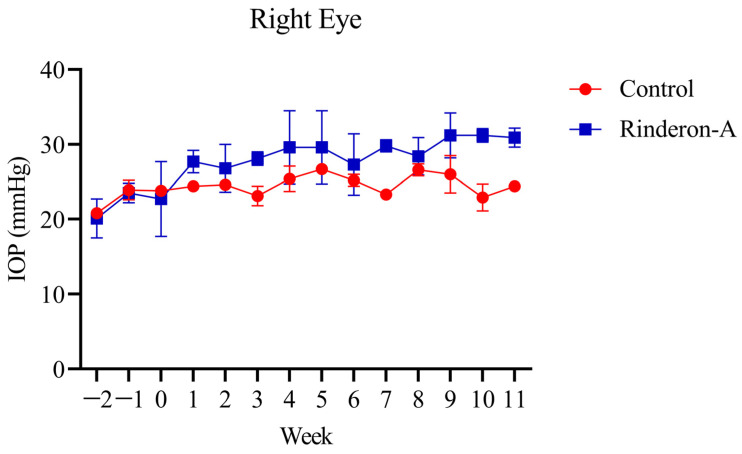
Trend of intra-ocular pressures of rats’ right eyes with or without Rinderon-A ointment application. Topical ocular normal saline (control) or Rinderon-A ointment was administered once daily over 11 weeks. The changes in IOP measurements of control and Rinderon-A ointment-treated eyes, indicating the mean IOP values 2 weeks before and 11 weeks after treatment. Values are present as mean ± SD from 5 measurements (*n* = 2–5). Analyzed by two-way mixed-effects model followed by Bonferroni’s post hoc testing.

**Figure 2:**
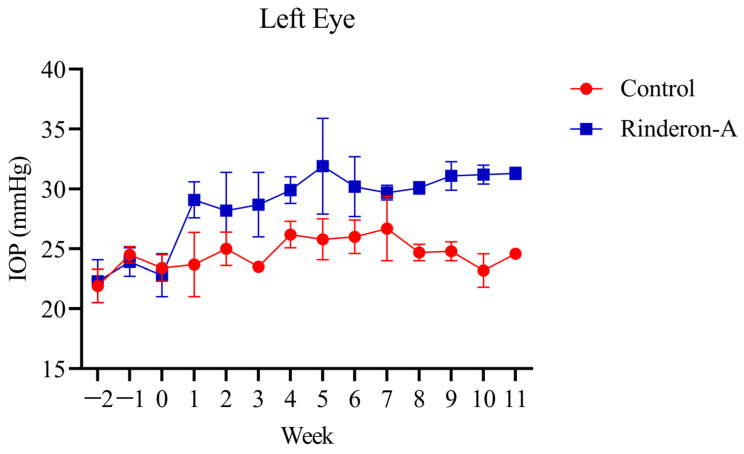
Trend of intra-ocular pressures of rats’ left eyes with or without Rinderon-A ointment application. Topical ocular normal saline (control) or Rinderon-A ointment was administered once daily over 11 weeks. The changes in IOP measurements of control and Rinderon-A ointment treated eyes, indicating the mean IOP values 2 weeks before and 11 weeks after treatment. Values are present as mean ± SD from 5 measurements (*n* = 2–5). Analyzed by two-way mixed-effects model followed by Bonferroni’s post hoc testing.

**Figure 3:**
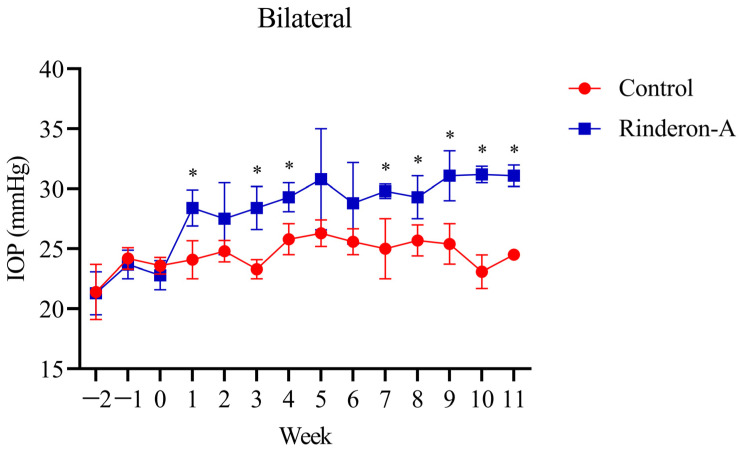
Trend of intra-ocular pressures of rats’ eyes with or without Rinderon-A ointment application. Topical ocular normal saline (control) or Rinderon-A ointment was administered once daily over 11 weeks. The changes in IOP measurements of control and Rinderon-A ointment treated eyes, indicating the mean IOP values 2 weeks before and 11 weeks after treatment. Values are presented as mean ± SD from 5 measurements (*n* = 2–5). * *p* < 0.05, compared with the control group. Analyzed by two-way mixed-effects model followed by Bonferroni’s post hoc testing. * *p* < 0.05 vs. control group.

Chronic elevation of IOP is believed to be a predominant risk factor for glaucoma; we therefore applied Rinderon-A ointment to rats’ eyes for the induction of chronically elevated IOP in a rat model. The mean values of IOP were recorded two weeks (W-2) before induction and after eleven weeks (W11) during Rinderon-A ointment treated periods (Table 1). As shown in Table 1, the normal IOP of a rat ranged from 21.3 ± 1.8 mmHg to 24.2 ± 0.9 mmHg (right eye: 20.1 ± 2.6 mmHg–23.9 ± 1.3 mmHg; left eye: 21.9 ± 1.4 mmHg–23.9 ± 1.2 mmHg, respectively) before Rinderon-A ointment application. No significant inter-group differences or time-by-group interactions were detected in either the right eye (F = 0.9666, *p* = 0.3386; Interaction: *p* = 0.920, Figure 1) or left eye (F = 2.635, *p* = 0.109; Interaction: *p* = 0.9223, Figure 2). In contrast, bilateral application of Rinderon-A produced a markedly ocular hypertensive response with a significant main effect for the treatment group (F = 44.03, *p* < 0.05) and longitudinal changes over time (F = 2.74, *p* = 0.05; Figure 3). Notably, the Group × Time interaction (F = 4.56, *p* < 0.05) confirms that Rinderon-A induced a time-dependent escalation of IOP over the experimental course, with post hoc multiple comparisons detecting significant differences between cohorts at Weeks 1, 3, 4, and 7–11 (*p* < 0.05).

## 4. Discussion

During the past years, rodent species have become of increasing interest in the study model of glaucoma. With recent advances, tonometry techniques provided an accurate measurement in small eyes, which might promote the research process in the animal model of glaucoma, especially in rodents [25]. In the present study, we established a simple and reliable method for inducing elevated IOP in rats through topical application of Rinderon-A ointment. This non-invasive and surgery-free approach provides several practical advantages over existing inducible ocular hypertension models, including anterior chamber injection of red blood cells or microbeads and episcleral vein obstruction, which require surgical procedures, specialized equipment, and technical expertise [26,27]. Therefore, this model may serve as a convenient alternative for longitudinal evaluation of corticosteroid-associated ocular hypertension. Consistent with the present findings, recent studies have also successfully applied steroid-induced mouse ocular hypertension models for longitudinal evaluation of intraocular pressure and pharmacological assessment of ocular hypotensive agents, further supporting the utility of corticosteroid-based experimental models for preclinical investigations [28]. Therapeutic uses of corticosteroids were estimated to be over 10 billion worldwide [29]. Notably, long-term corticosteroid treatment for autoimmune diseases and inflammatory disorders has been shown to frequently increase IOP, leading to ocular hypertension and glaucoma. The term corticosteroid-induced IOP elevation was first proposed as the arising prevalence of individuals that were susceptible to systemic administration and topical medication with cortisone [30,31]. Although steroid therapy has been reported to cause steroid-response ocular hypertension, emerging application of intravitreal use of triamcinolone acetonide to treat angiomatous proliferation and macular edema was observed to associate with a high incidence rate of induced ocular hypertension, all of which lack therapeutic options [32,33,34]. Thus, pharmacotherapeutic strategies for the treatment of drug-induced ocular hypertension and/or glaucoma on the basis of preclinical studies in animal models were necessary for modern drug discovery. In the present study, we established a simple and non-invasive rat model of corticosteroid-induced ocular hypertension using topical Rinderon-A ointment. This model provides an alternative to existing invasive ocular hypertension models while allowing longitudinal monitoring of IOP changes.

Although statistically significant differences were not consistently observed at every individual time point, topical Rinderon-A ointment produced a sustained increase in IOP throughout the 11-week experimental period. The IOP elevation was approximately 3–8 mmHg above that of the normal saline-treated eyes and reached a maximum mean value of 31.2 ± 0.7 mmHg. Rather than representing a transient increase, the persistent elevation in IOP suggests that repeated topical corticosteroid administration can establish a stable ocular hypertensive response under the present experimental conditions. These findings support the suitability of this model for longitudinal investigations of corticosteroid-associated ocular hypertension. The development and severity of corticosteroid-induced ocular hypertension are influenced by multiple factors, including corticosteroid formulation, administration route, dosing regimen, and genetic background. A recent dexamethasone-induced ocular hypertension study in C57BL/6J mice demonstrated that repeated steroid exposure increased IOP from approximately 11–12 mmHg to 22–23 mmHg, with peak elevation occurring around days 27–31, highlighting the impact of steroid exposure protocols on IOP dynamics [28]. In addition, comparative analyses among genetically distinct mouse strains showed that C57BL/6J and C3H/HeJ mice developed significant ocular hypertension following dexamethasone administration, whereas DBA/2J.Gpnmb+, 129P3/J, and BALB/cJ mice showed limited responses, suggesting that genetic background contributes to variability in corticosteroid sensitivity [20]. These findings suggest that differences in corticosteroid pharmacokinetics, tissue exposure, and intrinsic susceptibility among experimental animals may contribute to the heterogeneous pressure responses observed across ocular hypertension models. Therefore, careful characterization of experimental conditions, including steroid formulation and treatment schedule, is essential for establishing reproducible and interpretable models for glaucoma research. It should be noted that all IOP measurements were performed under ketamine/xylazine anesthesia, which has been reported to influence IOP in rodents. However, the same anesthetic regimen, dosage, and measurement protocol were applied consistently to all experimental groups throughout the study. Therefore, any potential effect of anesthesia on IOP was expected to be comparable across groups and is unlikely to have influenced the relative differences observed between treatments. Consistent with our findings, Zode et al. reported that topical dexamethasone administration induced ocular hypertension in mice, accompanied by retinal ganglion cell dysfunction and endoplasmic reticulum stress in ocular tissues [35]. Although mechanistic investigations were beyond the scope of the present study, both models demonstrated sustained corticosteroid-associated IOP elevation following topical corticosteroid exposure. In the present study, Rinderon-A ointment induced an increase in IOP as early as week 1 (28.4 ± 1.5 mmHg compared with 24.1 ± 0.6 mmHg in normal saline-treated eyes). Compared with the model reported previously [15], where IOP elevation was detected after a longer induction period, the earlier response observed in our model may be associated with differences in corticosteroid formulation and ocular exposure duration. In contrast to pharmacological induction models, primate ocular hypertension models commonly rely on laser photocoagulation to produce sustained IOP elevation. In these models, repeated laser procedures are generally required to achieve and maintain elevated IOP levels [28]. Although laser-induced models provide valuable information regarding glaucomatous progression, the invasive nature and technical requirements may limit their application in large-scale pharmacological screening. In comparison, the present Rinderon-A ointment-induced model allows non-invasive and repeated assessment of IOP changes over an extended period [36].

In addition to clinical observations in humans, topical corticosteroid administration has been widely used to establish experimental ocular hypertension in animal models. Lorenzetti first described a rabbit model of corticosteroid-induced ocular hypertension using repeated topical administration of several corticosteroids, including betamethasone 17-valerate, dexamethasone 21-phosphate, medrysone, and hydrocortisone, over a 12-week period [37]. In their work, dexamethasone 21-phosphate produced an IOP elevation of approximately 5–10 mmHg following twice-daily topical instillation. Compared with our rat model, topical application of Rinderon-A ointment once daily in the present study produced a sustained IOP increase of approximately 3–8 mmHg throughout the experimental period. Although the magnitude of IOP elevation was lower than that reported in rabbits, the once-daily ointment regimen successfully established a stable ocular hypertensive response. These differences may reflect species-specific responses, corticosteroid formulation, dosing frequency, and ocular drug retention. Razali et al. demonstrated that topical administration of 0.1% dexamethasone eye drops induced progressive ocular hypertension in rats, with approximately 80% of treated animals exhibiting a 36.9% increase in IOP after 62 days of treatment [38]. Shinzato et al. demonstrated that four weeks of topical dexamethasone treatment induced significant IOP elevation in rats [39]. Furthermore, both Shinzato et al. and Miyara et al. reported corticosteroid-associated alterations in the protein expression profiles of the trabecular meshwork and retina using proteomic analyses [39,40]. Collectively, these studies indicate that sustained ocular hypertension can be reproducibly established using different corticosteroid formulations and treatment protocols. However, variations in the onset, magnitude, and duration of IOP elevation among studies suggest that animal species, corticosteroid potency, dosage form, and experimental design all contribute to the observed ocular hypertensive responses. The differences in IOP elevation observed among corticosteroid-induced ocular hypertension models are likely multifactorial. It has been proposed that corticosteroid-induced ocular hypertension is primarily associated with increased resistance to aqueous humor outflow, a process that is generally reversible following withdrawal of corticosteroid treatment. Therefore, this experimental model is considered to mimic the physiological characteristics of corticosteroid-associated ocular hypertension rather than irreversible glaucomatous neurodegeneration. Taken together, the present findings demonstrate that topical application of Rinderon-A ointment provides a simple, non-invasive, and reproducible approach for establishing corticosteroid-associated ocular hypertension in rats. Compared with existing experimental models, this model facilitates longitudinal IOP monitoring without surgical intervention and may serve as a practical platform for evaluating potential therapeutic strategies for corticosteroid-associated ocular hypertension.

However, several limitations of the present study should be acknowledged. First and foremost, while our model successfully established steroid-induced ocular hypertension, critical parameters evaluating secondary glaucomatous neurodegeneration, such as RGC loss, optic nerve axonal density, retinal histology, functional OCT imaging, and inflammatory or fibrotic biomarkers, were not assessed in this initial phase. Therefore, this model currently represents a reliable rat model of steroid-induced ocular hypertension rather than a fully validated glaucoma model. Second, the exact mechanical pathways involving Rinderon-A ointment-induced IOP elevation were not fully elucidated, and longitudinal measurements regarding central corneal thickness, anterior scleral thickness, episcleral venous pressure, or potential corneal swelling were lacking, which constrained our ability to fully profile timeline responses during long-term treatment. Third, this study was designed as an exploratory pilot screening constrained by a small sample size and unequal group allocation (N = 10 total; *n* = 5, 3, and 2 rats per group). This unbalanced distribution was strictly bound by the 3Rs (Reduction) animal welfare principles to prioritize sample size for the primary induction cohort while maintaining the minimal necessary number for reference controls without animal mortality. Notably, a post hoc power analysis based on the primary longitudinal outcome (IOP elevation) demonstrated that the robust treatment effect yielded a large effect size (Cohen’s f > 0.80), achieving an adequate statistical power (1-β) exceeding 80% (α = 0.05). Potential statistical constraints were further addressed by utilizing a two-way mixed-effects model with Geisser–Greenhouse correction. Nevertheless, future investigations using larger, balanced cohorts and comprehensive neurodegenerative evaluations are warranted to validate and extend these preliminary findings.

## 5. Conclusions

The present ocular hypertensive rat model provided principal advantages of developing IOP elevation easily (mean IOP, 22.8 ± 1.2 mmHg to 31.2 ± 0.7 mmHg), a non-invasive technique without special equipment, and high cost-effectiveness of the experimental rats, which can be reliably manipulated in small eyes to investigate drug-induced ocular hypertension on the basis of therapeutic options.

## Figures and Tables

**Table 1:** Mean (SD) intraocular pressures of rats with or without application of drug.

Eyes	Group	W-2	W-1	W0 ^#^	W1	W2	W3	W4	W5	W6	W7	W8	W9	W10	W11
Right	Control	20.8 (0.0)	23.9 (1.3)	23.8 (0.0)	24.4 (0.6)	24.6 (0.3)	23.1 (1.3)	25.4 (1.7)	26.7 (0.4)	25.2 (0.8)	23.3 (0.4)	26.6 (0.8)	26.0 (2.5)	22.9 (1.8)	24.4 (0.0)
	Rinderon-A	20.1 (2.6)	23.5 (1.3)	22.7 (0.5)	27.7 (1.5)	26.8 (3.2)	28.1 (0.9)	29.6 (4.9)	29.6 (4.9)	27.3 (4.1)	29.8 (0.8)	28.4 (2.5)	31.2 (3.0)	31.2 (0.9)	30.9 (1.3)
	Statistics *	ns	ns	ns	ns	ns	ns	ns	ns	ns	ns	ns	ns	ns	ns
Left	Control	21.9 (1.4)	24.5 (0.7)	23.4 (1.1)	23.7 (2.7)	25.0 (1.4)	23.5 (0.1)	26.2 (1.1)	25.8 (1.7)	26.0 (1.4)	26.7 (2.7)	24.7 (0.7)	24.8 (0.8)	23.2 (1.4)	24.6 (0.3)
	Rinderon-A	22.3 (1.8)	23.9 (1.2)	22.8 (1.8)	29.1 (1.5)	28.2 (3.2)	28.7 (2.7)	29.9 (1.1)	31.9 (4.0)	30.2 (2.5)	29.7 (0.6)	30.1 (0.5)	31.1 (1.2)	31.2 (0.8)	31.3 (0.5)
	Statistics *	ns	ns	ns	ns	ns	ns	ns	ns	ns	ns	ns	ns	ns	ns
Bilateral	Control	21.4 (2.3)	24.2 (0.9)	23.6 (0.7)	24.1 (1.6)	24.8 (0.9)	23.3 (0.8)	25.8 (1.3)	26.3 (1.1)	25.6 (1.1)	25.0 (2.5)	25.7 (1.3)	25.4 (1.7)	23.1 (1.4)	24.5 (0.2)
	Rinderon-A	21.3 (1.8)	23.7 (1.2)	22.8 (1.2)	28.4 (1.5)	27.5 (3.0)	28.4 (1.8)	29.3 (1.2)	30.8 (4.2)	28.8 (3.4)	29.8 (0.6)	29.3 (1.8)	31.1 (2.1)	31.2 (0.7)	31.1 (0.9)
	Statistics *	ns	ns	ns	*	ns	*	*	ns	ns	*	*	*	*	*

Data are presented as mean ± SD. Statistical significance was assessed using a two-way mixed-effects model (REML) with Geisser–Greenhouse correction, followed by Bonferroni’s post hoc comparisons. * *p* < 0.05. ^#^ The first week of topical Rinderon-A ointment application. ns: not significance.

## Data Availability

The raw data supporting the conclusions of this article will be made available by the authors upon request.

## Abbreviations

The following abbreviations are used in this manuscript:

IOP	Intraocular pressure
RGCs	Retinal ganglion cells
